# Aging and neuronal death

**DOI:** 10.18632/aging.205433

**Published:** 2023-12-13

**Authors:** Fang Fang, Robert Usselman, Renee Reijo Pera

**Affiliations:** 1 The First Affiliated Hospital of USTC, Division of Life Sciences and Medicine, University of Science and Technology of China, China; 2 Chemistry Program, Department of Biomedical and Chemical Engineering and Sciences, Florida Institute of Technology, Melbourne, FL 32901, USA; 3 McLaughlin Research Institute, Great Falls, MT 59405, USA

**Keywords:** neurodegeneration, reactive oxygen species, aging, histone h3k79 methyltransferase, dopaminergic neurons

Neurons are among the most durable of cells in the body; they are born during embryonic development and must function in the body for the entire lifespan of the organism. Although they do not undergo replicative aging, neurons are subject to diverse sources of damage throughout life. Neurons may die of ischemia, as the activities of neurons require approximately 20 percent of the oxygen and 25 percent of the glucose that a body consumes. While consuming large amounts of oxygen, neurons can also accumulate damage, that may lead to cell death that is linked to reactive oxygen species (ROS). In addition, neurons might meet their demise due to ion overload and swelling due to the malfunction of voltage-gated ion channels on their membrane. More importantly, neurons may also die of high concentrations of neurotransmitters and accumulation of misfolded proteins, which have been observed in several neurodegenerative diseases [1].

**Figure 1:**
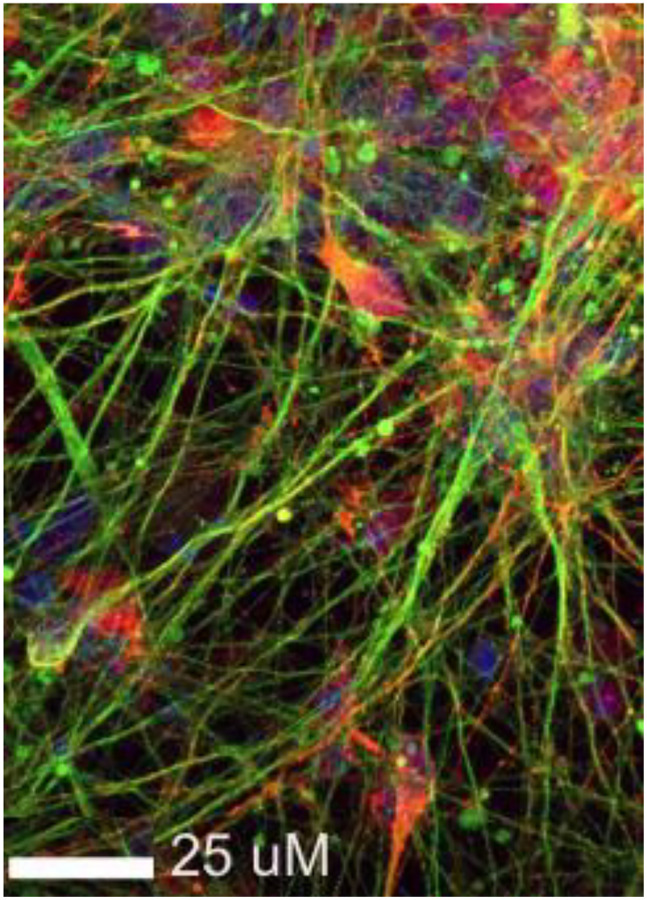
**Dopaminergic neurons differentiated from induced pluripotent stem cells *in vitro*.** Some processes that may contribute to neuron death can be assessed and modulated *in vitro* although those that are linked to aging are more difficult to replicate. Shown are neurons immunostained for BIII-tubulin (green) and alpha-synuclein (red).

Recently, Cui and colleagues described a high throughput screening method that used a previously characterized reporter construct consisting of the luciferase gene inserted downstream of the endogenous tyrosine hydroxylase (TH) gene and neurons differentiated from human pluripotent stem cells for 18 days to probe for factors that promote neuron differentiation and maintenance over time [2, 3]. Use of pluripotent stem cell-derived neurons may enable modeling of cell survival and demise under some conditions (Figure 1). The reporter mimicked expression of TH and activity could be measured non-invasively over time. Screening of the bioactive compound library resulted in the identification of a single molecule that is an inhibitor of a widely-conserved histone H3K79 methyltransferase that is able to both activate and repress gene transcription.

Additional screening tools, along with diverse cell-based, imaging and animal models, may enable us to address important questions such as what are the fundamental mechanisms that enable functional longevity of neurons? How might accumulation of ROS impact cell survival and death and how might accumulation be modulated to increase longevity? We know that ROS exert a wide range of biological effects from beneficial regulatory functions to deleterious oxidative stress when ROS are in excess of normal functional levels [4]. Moreover, research in ROS biology is beginning to highlight the distinction between global and local ROS balances and imbalances in cell phenotyping and mitochondrial energy management [5]. While global ROS homeostasis is important for the overall redox status of cells and tissues, ROS signaling pathways are thought to be driven locally by cellular microdomain-specific ROS production and degradation [6]. Local ROS production can be controlled by reduced flavoenzyme biochemical processes, such as substrate-induced enzyme regulation and alternative electron transport pathways in normal metabolism [7]. Further, to combat ROS, neurons express neurotrophic proteins (e.g., Engrailed) to enhance mitochondria’s activities by upregulating the expression of mitochondrial complex I [8]. A sustained disruption of ROS balance can result in desirable enhanced cell signaling or undesirable oxidative stress, which can either improve function or diminish performance, respectively. Similarly, it has been shown that neurons have developed a distinct transcriptome signature to repress genes related to neural excitation and synaptic function, extending longevity by preventing neurons from ion overload [9]. In addition, neurons have evolved specific DNA-repair mechanisms to repair errors induced by active transcription [10]. Finally, it is clear that avoiding apoptosis may contribute to long-term survival; neurons are capable of turning off pro-apoptotic genes by alternative splicing [11]. Together, these findings suggest that neurons have evolved a set of intrinsically interconnected mechanisms to reduce long-term accumulations of aging-related damages. Disruption in these mechanisms may tip the neuron homeostasis off-balance and drive the neurons into the path of degeneration. We have a plethora of tools to probe the fundamental mechanisms with hopes of translation to clinical applications.
